# Diversity of Purple Phototrophic Bacteria, Inferred from *pufM* Gene, within Epilithic Biofilm in Tama River, Japan

**DOI:** 10.1264/jsme2.ME11306

**Published:** 2012-03-23

**Authors:** Setsuko Hirose, Kenji V. P. Nagashima, Katsumi Matsuura, Shin Haruta

**Affiliations:** 1 Department of Biological Sciences, Tokyo Metropolitan University, Minami-Ohsawa 1–1, Hachioji, Tokyo, 192–0397, Japan

**Keywords:** Phototrophic bacteria, river, epilithic biofilm, *pufM*, purple bacteria

## Abstract

The diversity of purple phototrophic bacteria in algae-dominated biofilm of a streambed in Tama River, Japan was investigated. Clone library analysis of the *pufM* gene encoding a subunit of the photochemical reaction center of purple bacteria detected 18 operational taxonomic units (OTUs) in several classes of *Proteobacteria*. Most of the OTUs showed less than 85% identity to the PufM amino acid sequences of known phototrophic bacteria. These results suggest that phylogenetically divergent and unknown purple phototrophic bacteria are present in the epilithic biofilm of the river.

Purple phototrophic bacteria have been isolated from a variety of environments, such as sediments, soils and waters in ponds, lakes, lagoons and oceans (10, 11). As purple bacteria are metabolically versatile, *e.g.*, photosynthesis, degradation of organic compounds, nitrogen fixation or sulfide oxidation, they play important roles in ecosystems in the light.

Microbial ecological studies on freshwater environments have found several groups of purple phototrophic bacteria belonging to *Alpha-* or *Betaproteobacteria* from lakes (12, 17) and rivers (8, 9, 20, 23). Some of these purple bacteria are known to be anaerobic anoxygenic phototrophs. The presence of aerobic anoxygenic phototrophs (AAP), which carry out photosynthetic reactions only under aerobic conditions, has been also indicated by phylogenetic or physiological studies; however, the distribution and ecophysiology of purple phototrophic bacteria in freshwater environments has not been documented.

Epilithic biofilm is important to sustain the ecosystem in freshwater environments in terms of producing organic substrates, feeding animals and degrading organic matter. The streambed biofilm is known to be mainly composed of oxygenic phototrophs, *i.e.*, cyanobacteria and algae (2); however, no study has targeted purple phototrophic bacterial diversity in river biofilm. Dense assemblages of bacterial cells and their active respiration in biofilm possibly develop some anaerobic niches (6) even when phototrophs emit oxygen, and consequently both aerobic and anaerobic anoxygernic phototrophs may find their niches within river biofilm.

In this study, we applied a culture-dependent method and a molecular method based upon the *pufM* gene encoding a subunit of the photochemical reaction center to investigate the diversity of purple phototrophic bacteria in epilithic biofilm in an upstream region of a river where the amount of dissolved organic matter is limited. Phylogenies of *pufM* gene sequences are mostly consistent with those of the 16S rRNA gene (14), and thus the *pufM* gene is frequently utilized for genetic surveys of anoxygenic phototrophs (1, 4, 12, 16, 24).

Submerged cobbles of about 15 to 25 cm in longest length were collected from a streambed in riffle located in the upstream region of Tama River in Ohme City (35°47′13″N, 139°15′15″E), in the western suburbs of Tokyo, Japan in August 2009. The riffle width at the sampling site was 40 m. The water depth of the sampling site was about 20 cm. Water temperature, pH, biochemical oxygen demand (BOD) and flow velocity of the river water at the sampling time were 18°C, 7.6, 0.5 mg L^−1^ and 0.4 m s^−1^, respectively. Average values of dissolved oxygen, total nitrogen and total phosphorus in this region in July to September 2009 were 8.9±0.3 mg L^−1^, 0.79±0.10 mg L^−1^ and 0.017±0.005 mg L^−1^, respectively (monthly report by Bureau of Environment, Tokyo Metropolitan Government, http://www.kan-kyo.metro.tokyo.jp). A brownish biofilm of about 1 mm thickness was present on the cobbles. A total 150 cm^2^ area of epilithic biofilm was scraped off from the top surface of each cobble using a sterile toothbrush and suspended into 10 mL sterile distilled water.

For bacterial culture, 0.1 mL of the biofilm suspension was transferred into a 30 mL volume screw cap tube filled with PE medium (7), a semisynthetic medium containing organic compounds. The tubes were incubated at 30°C under filtered incandescent light (ca. 2,000 lux) of wavelength over 700 nm for 7 to 14 days. The cultures which showed spectral properties of purple bacteria were streaked on agar plates of PE medium. The plates were incubated anaerobically under incandescent light and red colonies were transferred and streaked onto new plates. These operations were repeated more than two times to obtain pure cultures.

Total genomic DNAs were directly extracted from the collected biofilm according to Noll *et al.*(15). DNA fragments of the *pufM* gene coding for the M subunit of the photochemical reaction center were amplified. Nested PCR was conducted to amplify *pufM* gene fragments from environmental DNA using primer sets pufLM-F/pufLM-R (1st PCR) (14) and M150f/M572r (2nd PCR) (16). PCR products (approximately 380 bp) were cloned with the pTAC-1 Vector (DynaExpress TA cloning kit, BioDynamics Laboratory, Tokyo, Japan). *Escherichia coli* JM109 competent cells (Nippon Gene, Tokyo, Japan) were transformed according to the manufacurer’s instructions. DNA sequences were determined with the BigDye v3.1 cycle sequencing kit (Applied Biosystems, Foster City, CA, USA) and a DNA sequencer ABI3130xl (Applied Biosystems). Chimeric clones were checked manually and excluded from further analyses. The phylogenetic tree based on the amino acid sequences of the partial PufM was constructed using the neighbor-joining and maximum-likelihood methods with the MEGA version 5 program (19, 22).

Cultivation of the river biofilm on PE medium found red-brown colonies, all of which were similar in morphology. Among them, 6 strains were isolated to determine the *pufM* sequences. Sequence analysis indicated that the isolates were classified into two, one of which was designated Tisolate 25 and the other Tisolate 231.

Figure 1 shows a neighbor-joining tree based on the amino acid sequences suspected from the partial *pufM* gene sequences from 37 clones and two isolates obtained in this study together with those from the database. A phylogenetic tree using the maximum-likelihood method showed tree topology roughly consistent with that in Fig. 1 (data not shown). Two major clades were recognized; one containing alpha-1, alpha-2, alpha-4 subclasses, beta and gamma classes of *Proteobacteria* and the other containing the alpha-3 subclass of *Proteobacteria*. This was roughly in agreement with earlier studies concerning *pufLM* or *pufM* phylogeny (4, 14). Obtained clones were grouped into 18 operational taxonomic units (OTUs). Each OTU was defined as a group having amino acid sequence identities above 90%. These OTUs were widely distributed in the phylogenetic tree and many were distantly related. No dominant OTUs in terms of numbers of clones were found, since every OTU consisted of less than 5 clones.

OTUs except OTU 6 showed less than 85% identity to the PufM sequences of the cultivated bacteria in the database. Sequences of OTU 6, which has the same sequence as that of Tisolate 25, was closely related to that of *Rhodoferax fermentans* (accession no. D50650, 98.4% identity). The other isolate, Tisolate 231, had 100% PufM sequence identity to *Rhodopseudomonas palustris* (accession no. AB015977). *R. fermentans* and *R. palustris* are known to be anaerobic anoxygenic phototrophs (11). Tisolate 25 and Tisolate 231 were grown photoheterotrophically under anaerobic conditions.

The alpha-3 clade contains 6 OTUs (OTUs 13 to 18). OTU 13 is distantly related to other members in this clade. Sequences included in OTU 14 showed very low identities to those of the database; the highest identity was 65.7% to that of an environmental clone (accession no. AY912082) (23) collected from river water. OTUs 15 to 18 formed a clade with *Staleya guttiformis* (now known as *Sulfitobacter guttiformis*) and alpha proteobacterium R2A163 and R2A84 (21), reported as aerobic anoxygenic phototrophic bacteria isolated from saline environments.

OTUs 1 and 2 were related to *Rhodospirillum rubrum*, belonging to alpha-1 subclass of *Proteobacteria*, with 75.4% and 71.4% sequence identities, respectively. OTU 3 was found to be similar to a marine gamma proteobacterium (5) with 84.1% sequence identity. OTU 4 was related to *Methylocella* sp. and *Rhodoplanes* sp. belonging to alpha-2 subclass of *Proteobacteria*. OTU 5 was similar to *R. fermentans* with 73.0% identity. It is indicated that OTUs 7 to 11 were grouped with the genus *Sphingomonas*, *Citromicrobium*, *Erythorobacter* and *Porphyrobacter* belonging to alpha-4 subclass. Phototrophic bacteria in this subclass have been known to be aerobic anoxygenic phototrophs. OTU 12 showed low identities to known sequences, and a close relative was *Halorhodospira halophila*, belonging to *Gammaproteobacteria* (69.0% identity).

In this study, we investigated the diversity of purple phototrophic bacteria in a streambed biofilm. Co-occurrence of possibly aerobic (*e.g.*, OTUs 15 to 18) and anaerobic (*e.g.*, OTU 6 and Tisolate 231) anoxygenic phototrophs was observed within the river biofilm as expected. Most OTUs detected by *pufM* clone library analysis had low identities to the sequences of cultured bacteria. Studies on bacterial communities in river biofilms using 16S rRNA gene analyses have also detected many clones of uncultured bacteria (3, 8, 13).

Phylogenetic analysis of the PufM sequences indicated that purple phototrophic bacteria in the river biofilm are widely distributed to alpha subclasses, beta and gamma classes of *Proteobacteria*. Such high diversity of purple phototrophic bacteria has not been reported in other environments. In French Mediterranean coast lagoon sediments, *pufM* clones in alpha-3 subclass of *Proteobacteria* accounted for 94.9% of total *pufM* clones (18). In the case of antarctic lake water, no *pufM* clones were related to alpha-3 subclass of *Proteobacteria* but 80% of the *pufM* clones were related to *Betaproteobacteria*(12). Microenvironments of algae-dominated river biofilm under the shallow and rapid flow of water are highly heterogeneous, differing in concentrations of dissolved oxygen, organic and inorganic compounds. The population of purple phototrophic bacteria within the epilithic biofilm microflora may be low because a nested approach was required to amplify their DNAs. The niche for purple phototrophic bacteria in the biofilm may be restricted, but seem to have largely diverged.

As purple phototrophic bacteria have bacteriochlorophylls, which have absorption bands at different wavelengths from those of chlorophylls in oxygenic phototrophs, purple photorophic bacteria can capture light energy even in algae-dominated biofilms of rivers. In the biofilm community, in addition to the primary production and degradation of organic matter, some purple phototrophic bacteria possibly contribute to oxidize sulfide produced by sulfate-reducing bacteria, since we observed sulfide production from anaerobic culture of the epilithic biofilm used in this study when illumination was stopped (data not shown).

This study demonstrated the unexpected diversity of purple photophototrophic bacteria in river biofilm. Physiological studies of yet-to-be cultured epilithic purple phototrophic bacteria will clarify the roles of these bacteria in the river ecosystem.

The nucleotide sequences determined in this study have been deposited in the GenBank/EMBL/DDBJ database under accession numbers AB670200 to AB670233.

## Figures and Tables

**Fig. 1:**
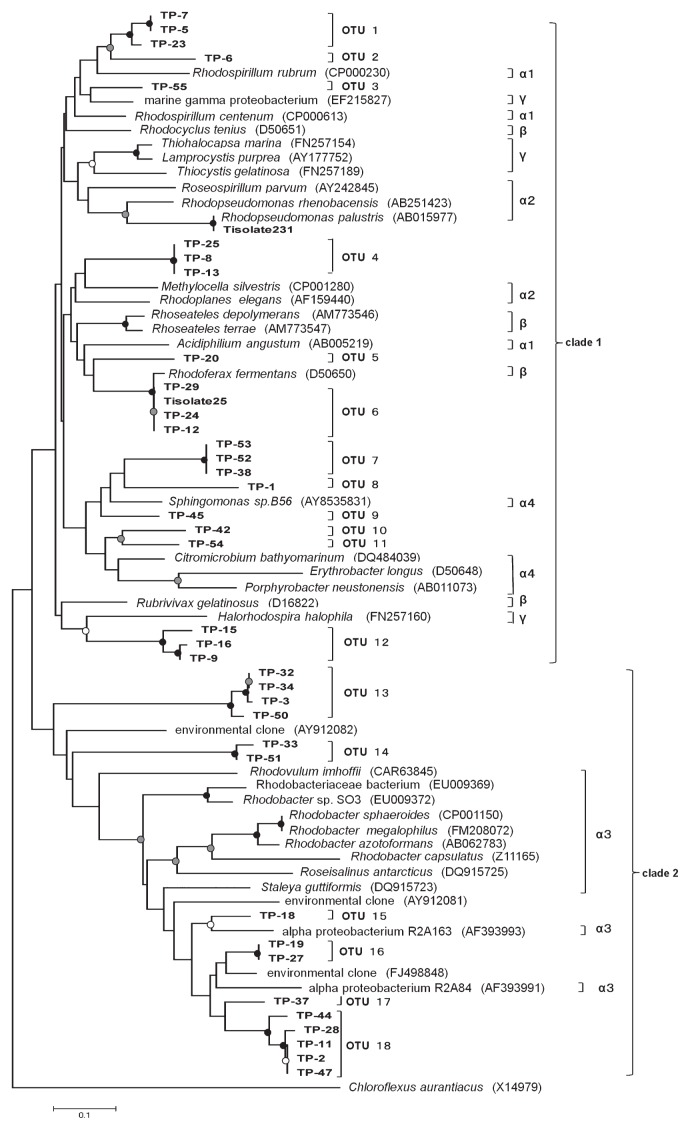
Phylogenetic tree of partial PufM amino acid sequences inferred from gene sequences. *Chloroflexus aurantiacus* was used as an outgroup. PufM amino acid sequences from environmental DNA in this study are indicated by TP-1–TP-55, and those from isolates in this study are indicated by the ‘Tisolate’ prefix. Sequences from the database are represented with their respective accession numbers after bacterial names in parentheses. OTUs are indicated to the right of the tree. Alpha-1, alpha-2, alpha-3, alpha-4 subclass, beta and gamma class of *Proteobacteria* are also indicated by α1, α2, α3, α4, β and γ to the right of the tree. Bootstrap values >90, 70–89 and 50–69% are indicated by black, gray and open circles, respectively. Scale bar represents the number of substitutions per site.
